# Distinct Effects of Different Phosphatidylglycerol Species on Mouse Keratinocyte Proliferation

**DOI:** 10.1371/journal.pone.0107119

**Published:** 2014-09-18

**Authors:** Ding Xie, Mutsa Seremwe, John G. Edwards, Robert Podolsky, Wendy B. Bollag

**Affiliations:** 1 Charlie Norwood VA Medical Center, Augusta, Georgia, United States of America; 2 Department of Physiology, Medical College of Georgia at Georgia Regents University, Augusta, Georgia, United States of America; 3 Apeliotus Technologies, Inc., Atlanta, Georgia, United States of America; 4 Center for Biotechnology and Genomic Medicine, Department of Medicine, Medical College of Georgia at Georgia Regents University, Augusta, Georgia, United States of America; University of Tennessee, United States of America

## Abstract

We have previously shown that liposomes composed of egg-derived phosphatidylglycerol (PG), with a mixed fatty acid composition (comprising mainly palmitate and oleate), inhibit the proliferation and promote the differentiation of rapidly dividing keratinocytes, and stimulate the growth of slowly proliferating epidermal cells. To determine the species of PG most effective at modulating keratinocyte proliferation, primary mouse keratinocytes were treated with different PG species, and proliferation was measured. PG species containing polyunsaturated fatty acids were effective at inhibiting rapidly proliferating keratinocytes, whereas PG species with monounsaturated fatty acids were effective at promoting proliferation in slowly dividing cells. Thus, palmitoyl-arachidonyl-PG (16∶0/20∶4), palmitoyl-linoleoyl-PG (16∶0/18∶2), dilinoleoyl-PG (18∶2/18∶2) and soy PG (a PG mixture with a large percentage of polyunsaturated fatty acids) were particularly effective at inhibiting proliferation in rapidly dividing keratinocytes. Conversely, palmitoyl-oleoyl-PG (16∶0/18∶1) and dioleoyl-PG (18∶1/18∶1) were especially effective proproliferative PG species. This result represents the first demonstration of opposite effects of different species of a single class of phospholipid and suggests that these different PG species may signal to diverse effector enzymes to differentially affect keratinocyte proliferation and normalize keratinocyte proliferation. Thus, different PG species may be useful for treating skin diseases characterized by excessive or insufficient proliferation.

## Introduction

Keratinocyte proliferation and differentiation are precisely regulated processes which are essential for proper formation and function of the epidermis of the skin to serve as a physical and water-permeability barrier [1], [2]. Because this largest organ of the body serves as the interface between the internal and external environments, the skin senses and responds to a variety of stresses (reviewed in [3]). Defects in the regulation of this growth/differentiation program, and the epidermis’ inability to restore homeostasis when stressed, can result in an abnormal barrier and a variety of skin diseases, such as non-melanoma skin cancer and psoriasis [4].

Previously, our laboratory has shown the existence of a novel cell signaling module composed of the glycerol transporter, aquaporin-3 (AQP3) and phospholipase D2 (PLD2). Phospholipase D (PLD) is a lipid-metabolizing enzyme that can catalyze both phospholipid hydrolysis to produce phosphatidate and a transphosphatidylation reaction using primary alcohols, such as glycerol, to generate phosphatidylalcohols [5]. In addition, we showed that PLD2, one isoform of PLD, colocalizes with AQP3 in, and co-immunoprecipitates from, caveolin-rich membrane microdomains in epidermal keratinocytes [6]. Together these two proteins appear to function to produce phosphatidylglycerol (PG) [7], which is important in the regulation of keratinocyte function [5], [6], [8], [9]. Indeed, manipulating this novel signaling module, the AQP3/PLD2/PG unit, alters keratinocyte proliferation and differentiation [8]. For instance, direct provision of liposomes produced from egg-derived PG (egg PG) results in an inhibition of keratinocyte proliferation in rapidly dividing keratinocytes [8]. Interestingly, however, in slowly dividing cells egg PG liposomes stimulate proliferation, suggesting that egg PG can normalize keratinocyte function [8]. Although there are many questions remaining to be answered about this novel cell signaling module, the ability of egg PG to normalize keratinocyte function is of interest because of the wide range of possible clinical applications to skin diseases characterized by abnormal proliferation and the potential for targeting this PLD2/AQP3/PG signaling modue for their treatment.

Egg PG is comprised of multiple PG species, with different acyl groups identifying the different PG species. Thus, egg PG exhibits the following fatty acid composition (with the first number representing the total number of carbon atoms in the fatty acid and the second number, the number of double bonds): 16∶0 (34%) 16∶1 (2%), 18∶0 (11%), 18∶1 (32%), 18∶2 (18%) and 20∶4 (3%) (Avanti Polar Lipids website). As a first step to define the mechanism underlying the normalization effect of egg PG, we sought to identify the PG species most effective at altering keratinocyte proliferation, with the assumption that the same species of PG would exert both effects on proliferation (inhibition of rapidly proliferating keratinocytes and enhancement of slowly growing cells). Cell proliferation was examined in order to screen a large number of PG species, although as the initial step in differentiation, growth arrest (or reversal of growth arrest) often reflects effects on other differentiation processes, such as involucrin levels as we have shown previously [8]. We found that various PG species affected keratinocyte proliferation differently; these results actually favor the potential clinical applications of different PG species for the treatment of different skin diseases, characterized by hyper- or hypoproliferation.

## Materials and Methods

### Materials

Dihexanoylphosphatidylglycerol (DHPG), dipalmitoylphosphatidylglycerol (DPPG), distearoylphosphatidylglycerol (DSPG), palmitoyl-oleoylphosphatidylglycerol (POPG), dioleoylphosphatidylglycerol (DOPG), palmitoyl-arachidonoylphosphatidylglycerol (PAPG), palmitoyl-linoleoylphosphatidylglycerol (PLPG), dilinoleoylphosphatidylglycerol (DLPG), soy-derived PG (soy PG), egg-derived PG, and dilinoleoylphosphatidylpropanol (DLPP) were all obtained from Avanti Polar Lipids, Inc. (Alabaster, AL). The composition of egg PG is provided in the Introduction. Soy PG is composed of 16∶0 (17%), 18∶0 (6%), 18∶1 (13%), 18∶2 (59%), and 18∶3 (5%) (Avanti Polar Lipids website). Calcium-free minimal essential medium and antibiotics were obtained from Biologos, Inc. (Maperville, Illinois). Bovine pituitary extract and epidermal growth factor were purchased from Life Technologies, Inc. (Grand Island, New York). ITS+(6.25 µg insulin per mL, 6.25 µg transferrin per mL, 6.25 ng selenous acid per mL, 5.35 mg linoleic acid per mL, and 0.125% bovine serum albumin) was supplied by Collaborative Biomedical Products (Bedford, Massachusetts).

### Keratinocyte Preparation and Cell culture

All animal studies were performed under a protocol approved by the Georgia Regents Institutional Animal Care and Use Committee and adhere to the standards described in the Guide for the Care and Use of Laboratory Animals. Mouse epidermal keratinocyte cell cultures were prepared from ICR strain CD-1 outbred neonatal mice 1–3 days of age as described in detail in [10], [11]. Briefly, the skins were harvested and incubated overnight in 0.25% trypsin at 4°C. The epidermis and dermis were separated and the basal keratinocytes scraped from the underside of the epidermis. The cells were collected by centrifugation and incubated overnight in an atmosphere of 95% air/5% CO_2_ at 37°C in plating medium [5]. The cells were refed every 1–2 days with serum-free keratinocyte medium (SFKM) also as in [5] until use. The newborn mice used for preparation of primary keratinocytes were anethetized by hypothermia and euthanized by decapitation. All procedures were conducted in conformity with the Public Health Service Policy on Humane Care and Use of Laboratory Animals and approved by the Institutional Animal Care and Use Committee.

### DNA Synthesis Assay

[^3^H]Thymidine incorporation into DNA was assayed as a measure of keratinocyte proliferation as described in [12]. Briefly, keratinocyte cultures were incubated for 24 hours in SFKM containing various concentrations of different PG species or DLPP, prepared as liposomes by sonication. [^3^H]Thymidine at a final concentration of 1 µCi/mL, was added to the cells for an additional 1-hour incubation. Reactions were terminated using ice-cold 5% trichloroacetic acid and unincorporated radiolabel removed by washing. Cells were solubilized in 0.3 M NaOH, and the radioactivity incorporated into DNA quantified by liquid scintillation spectroscopy.

### Statistical Analysis

Data are expressed as mean ± SEM. Experiments were performed a minimum of three times and analyzed by ANOVA with a Student-Newmann-Keuls or Dunnett’s post-hoc test using Instat or Prizm software (Graphpad, La Jolla. CA). *P*≤0.05 was considered statistically significant.

To compare the different effects of all of the PGs investigated together, it was necessay to use a mixed model analysis of variance to analyze the effects in different experimental settings. Prior to analysis to improve assumptions of normality and additivity for the models to be used, the data were transformed and grouped. Because one of the treatments had 0 concentration units, we transformed concentration using ln[PG concentration + exp(2.5)], which results in even spacing of the transformed concentrations. Cell proliferation rate (counts per minute; CPM) was transformed using ln(CPM). Because in some experiments cells were quickly proliferating, while in other experiments the cells were not, we grouped experiments that had different responses to egg PG using finite mixture regression models and the flexmix package detailed in [13]. This method clusters each experiment by the regression fit between ln(CPM) and ln(concentration), grouping cultures with similar regression lines in the same cluster. Because the relationship between ln(CPM) and ln(concentration) was not linear, we used quadratic regression for the clustering analysis. The number of clusters was determined using the Bayesian information criterion (BIC). This analysis initially suggested that eight groups of regression lines were present for the egg PG data. Inspection of plots of these groups indicated four patterns were present with some variation within these four patterns, based on the relationship between ln(CPM) and ln(concentration) of egg PG: (1) a linear increasing relationship; (2) a non-linear decreasing relationship; (3) a flat, quadratic relationship; and (4) a decreasing step function relationship. These four general patterns were used as the four groups in subsequent analyses. While the data do support eight clusters, collapsing these clusters together does not affect the subsequent analyses that examined the effects of different PGs on the relationship between ln(CPM) and ln(concentration), since the differences in slopes and intercepts within a cluster can be accommodated within the analyses.

We used a mixed model analysis of variance separately for each of the four grouped clusters identified above to determine how the different PG species affected the relationship between cell proliferation and PG concentration. The model used for this analysis of variance included PG species as a fixed effect that potentially interacted with ln(concentration), and random coefficients for each culture/experiment. This analysis accounts for the different responses for each individual culture/experiment. We focused on three effects from this model: (1) the PG species effect, which reflects the PG species change in intercept; (2) the PG species by ln(concentration) effect, which reflects the PG species change in the linear slope; and (3) the PG species by ln(concentration)^2^ effect, which reflects the PG species change in the quadratic regression coefficient. The first effect relates to the overall difference in means between the PG species, although this difference may be dose dependent as reflected by the last two effects. Together, these three effects reveal how PG species change the overall relationship between cell proliferation [ln(CPM)] and PG concentration. The mixed model analysis was conducted using the Proc Mixed procedure in SAS 9.13. *P*≤0.05 was deemed as significant.

## Results

### PG species containing saturated or monounsaturated fatty acids stimulated keratinocyte proliferation

**Figure 1:**
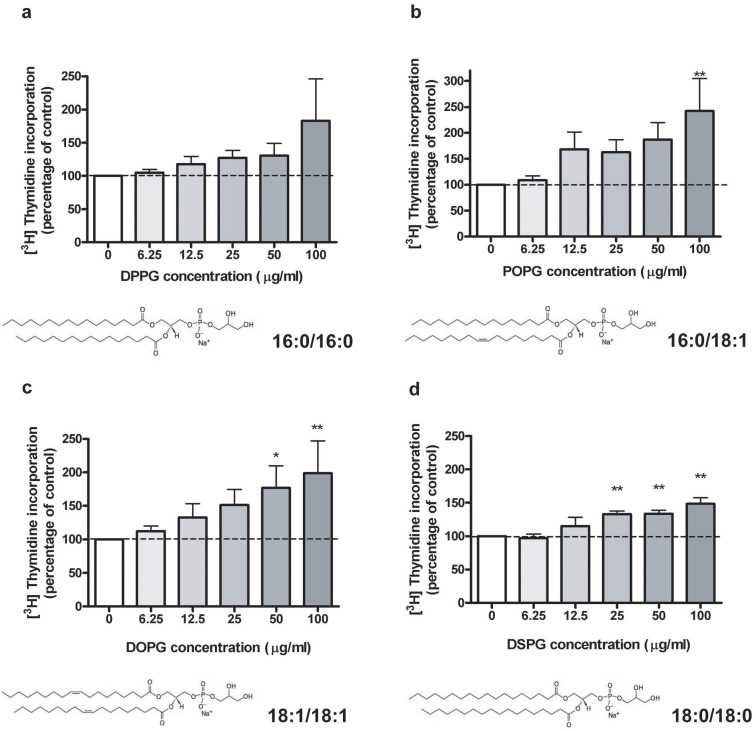
Effects of PG species containing saturated or monounsaturated fatty acids on keratinocyte proliferation. Near-confluent keratinocytes were treated for 24****hrs with the indicated concentrations of liposomes of DPPG (A), POPG (B), DOPG (C) or DSPG (D), prepared via bath sonication of the different PG species in SFKM. [^3^H]Thymidine incorporation into DNA was then determined as in [10]. Values represent the means ± SEM of 4 to 11 separate experiments performed in duplicate; *p<0.05, **p<0.01 versus the control value (0** µ**g/mL). [^3^H]Thymidine incorporation into DNA in the control was 31,300±8,400 cpm/well, 33,600±9,800 cpm/well, 32,200±8,700 cpm/well, and 44,200±1,200 cpm/well for panels A, B, C and D, respectively.

Since previous data indicated an ability of egg PG to inhibit the proliferation of rapidly dividing and stimulate the growth of slowly proliferating keratinocytes [14], we first examined the effect on keratinocyte proliferation of the PG species containing the most abundant acyl groups found in egg PG. Since the major acyl groups associated with egg-derived lipids are palmitic acid (16∶0), and oleic acid (18∶1), the synthetic PGs, DPPG (16∶0/16∶0), POPG (16∶0/18∶1), and DOPG (18∶1/18∶1) were initially chosen to examine their effects on the proliferation of primary cultures of mouse epidermal keratinocytes. PG prepared in the form of liposomes was provided to the keratinocytes at doses ranging from 0 to 100 µg/mL. In all cases an egg PG comparator dose response was performed for comparison among experiments of the effects of different PG species. POPG and DOPG significantly stimulated DNA synthesis in a dose-dependent manner (Figure 1B and 1C). The initial dose of POPG and DOPG demonstrating a significant stimulatory effect was 100 µg/mL and 50 µg/mL, respectively. Although there was no significant effect of DPPG on DNA synthesis, it followed the same trend (Figure 1A). Contrary to our assumption that a certain PG species would exert both inhibitory and stimulatory effects, these results suggested that some PG species might stimulate keratinocyte proliferation while others might inhibit keratinocyte proliferation. In addition, our data suggested that the monounsaturated fatty acid tails in PG may play a role in this stimulatory effect. We also determined that 100 µg/mL DSPG (18∶0/18∶0) significantly increased [^3^H]thymidine incorporation by 36%, suggesting a likely pro-stimulatory effect of saturated fatty acids as well (Figure 1D). Since none of the PG species containing the most abundant fatty acids in egg PG inhibited keratinocyte proliferation, we opted to test additional PG species, containing fatty acids representing a minor fraction of those comprising egg PG.

### PG species containing polyunsaturated fatty acids inhibited keratinocyte proliferation

**Figure 2:**
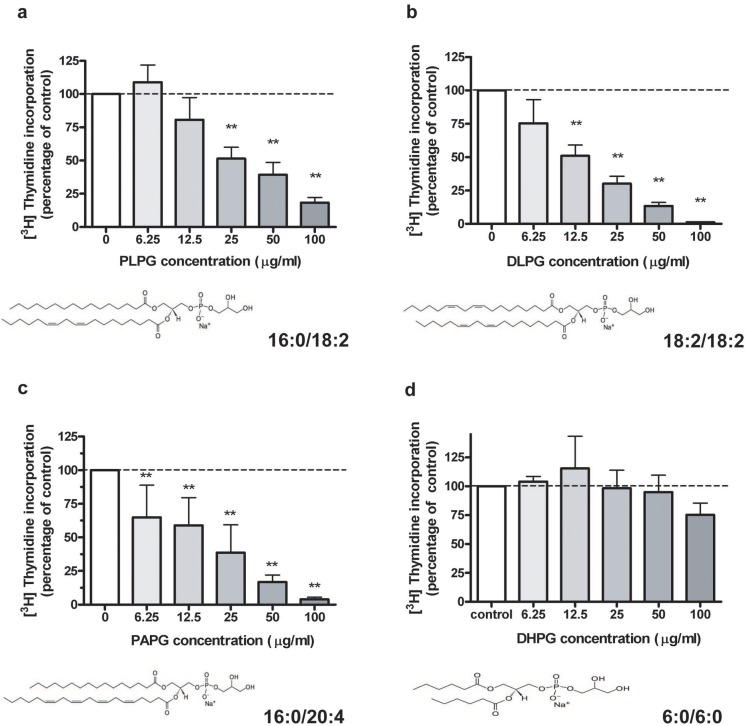
Effects of PG species containing polyunsaturated fatty acids on keratinocyte proliferation. Near-confluent keratinocytes were treated for 24 hrs with the indicated concentrations of liposomes of PLPG (A), DLPG (B), PAPG (C) or DHPG (D), prepared via bath sonication of the different PG species in SFKM. [^3^H]Thymidine incorporation into DNA was then determined as above. Values represent the means ± SEM of 3 to 5 separate experiments performed in duplicate; *p<0.05, **p<0.01 versus the control value (0 µg/mL level served as control in each panel). [^3^H]Thymidine incorporation into DNA in the control was 45,300±4,900 cpm/well, 37,700±8,900 cpm/well, 66,000±13,000 cpm/well, and 55,300±8,900 cpm/well for panels A, B, C, and D respectively.

PLPG (16∶0/18∶2), DLPG (18∶2/18∶2), and PAPG (16∶0/20∶4), each possessing at least one polyunsaturated fatty acid, were selected as PG species containing fatty acids comprising a minor fraction of those in egg PG. These PG species, prepared as liposomes, were directly provided to the primary mouse keratinocytes at doses as described above. Synthetic PLPG, DLPG, and PAPG were particularly effective at inhibiting keratinocyte proliferation in a dose-dependent manner (Figure 2A, B, and C). The initial dose of PLPG, DLPG, and PAPG demonstrating a significant inhibitory effect was 25 µg/mL, 12.5 µg/mL, and 6.25 µg/mL, respectively. Note the apparent trend of increasing inhibitory effects with a greater degree of (poly)unsaturation.

We also tested a synthetic PG with short acyl chains, which should be more soluble in aqueous solution and thus more easily applied, and determined that there was no significant effect of DHPG (6∶0/6∶0) on keratinocyte proliferation (Figure 2D). This result suggests that the natural, longer chain fatty acid-containing PGs may be more effective in regulating keratinocyte function.

### Soy PG inhibited keratinocyte proliferation

**Figure 3:**
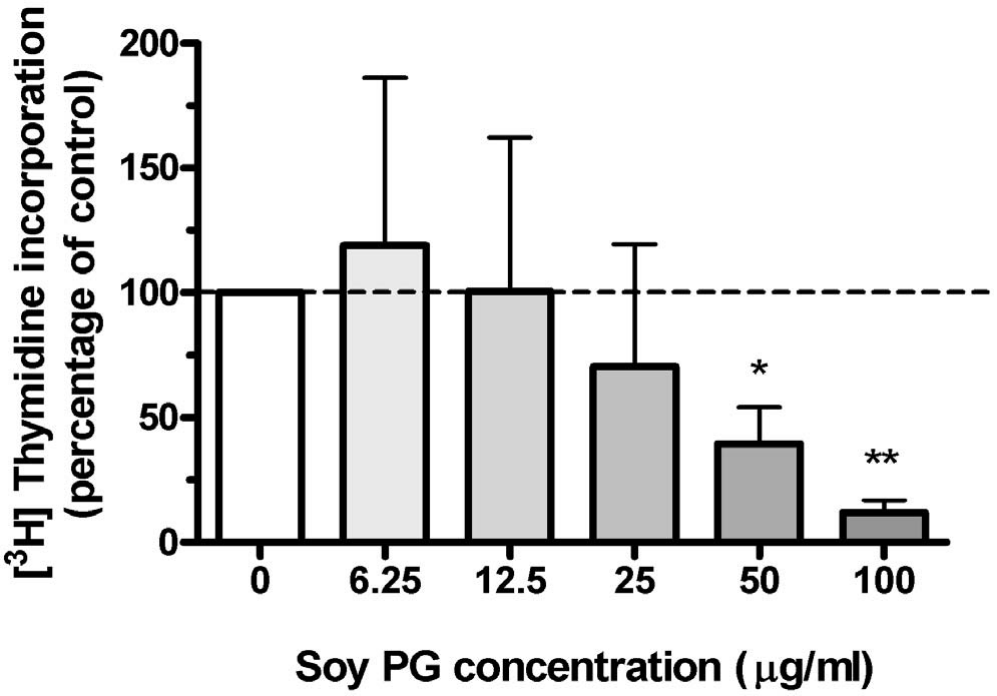
Effects of soy PG on keratinocyte proliferation. Near-confluent keratinocytes were treated for 24 hrs with the indicated concentrations of soy PG liposomes, prepared via bath sonication of the phospholipid in SFKM. [^3^H]Thymidine incorporation into DNA was then determined as above. Values represent the means ± SEM of 5 separate experiments performed in duplicate; *p<0.05, **p<0.01 versus the control value (0 µg/). [^3^H]Thymidine incorporation into DNA in the control was 62,600±8,000 cpm/well.

Our results with PLPG, DLPG, and PAPG suggested an important role of polyunsaturated fatty acids in PG’s antiproliferative effects. However, these synthetic PGs are expensive to produce. Considering the potential medical application of this phospholipid to hyperproliferative skin disorders, we sought to determine the effects on keratinocyte proliferation of soy PG, which is a less expensive PG mixture containing a large percentage of polyunsaturated fatty acids, including linoleic (18∶2) and linolenic (18∶3) acids (13% and 59%, respectively). Consistent with the effects of synthetic PG species containing polyunsaturated fatty acids, soy PG also significantly inhibited DNA synthesis in a dose-dependent manner (Figure 3). We next sought to determine the most effective of these tested PG species on keratinocyte proliferation in comparison to egg PG for use in further experiments to test potential medical applications.

### Comparison of the effects of different PG species on keratinocyte proliferation

**Figure 4:**
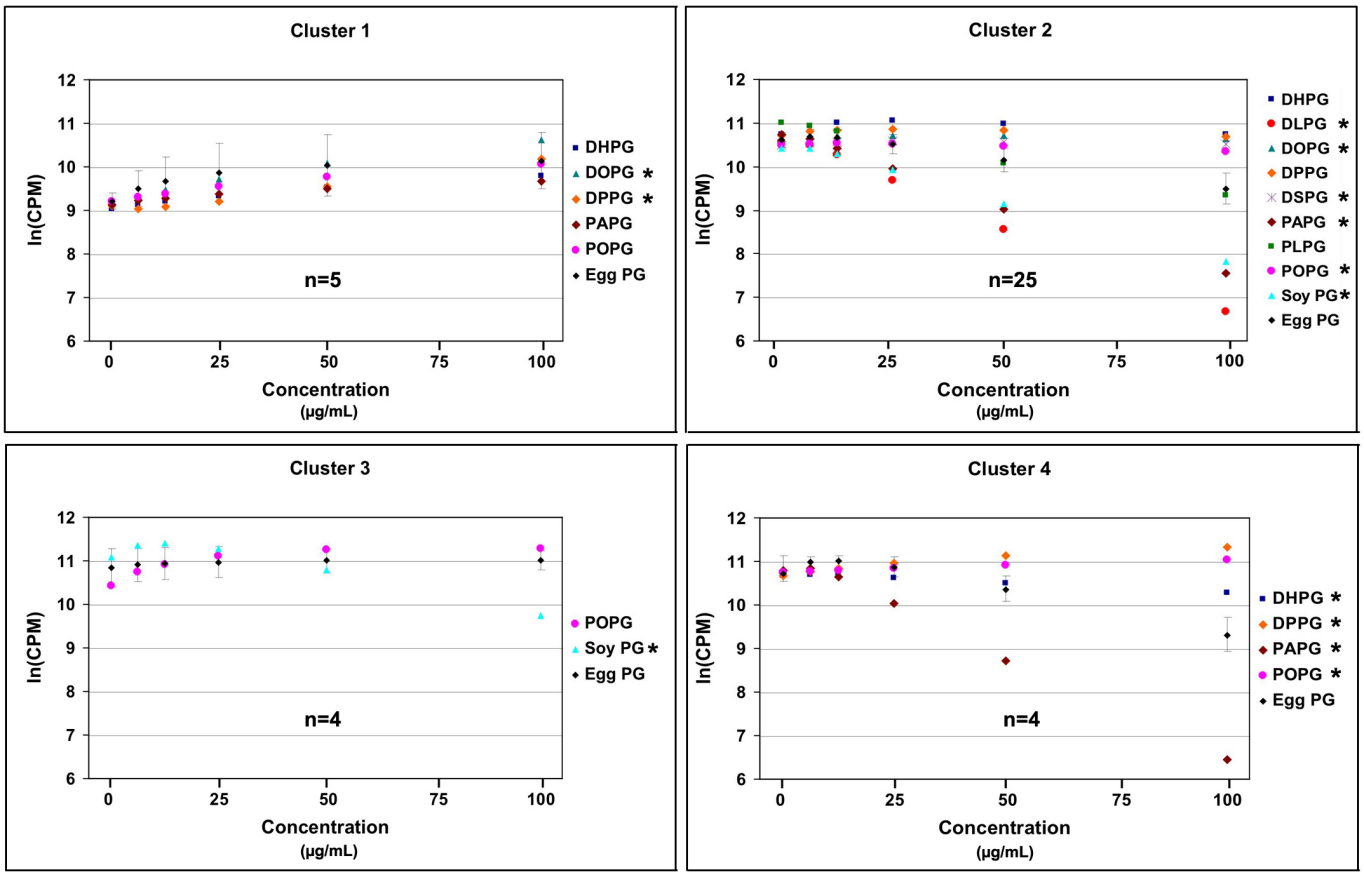
Comparison of Effects of PG Species on Keratinocyte Proliferation. Based on the regression line of the comparative egg PG data, Cluster 1 represents the experiments with a linear increasing relationship between ln(CPM) and ln(concentration) of egg PG, Cluster 2 represents the experiments with a non-linear decreasing relationship between ln(CPM) and ln(concentration), Cluster 3 represents the experiments with a flat, quadratic relationship between ln(CPM) and ln(concentration), and Cluster 4 represents the experiments with a decreasing step function between ln(CPM) and ln(concentration), respectively. Those PG species exhibiting a statistically significant difference from egg PG (either a greater or lesser effect) are marked by asterisks. The number of different experiments for which the egg PG comparator was performed is indicated.

It was not technically feasible to conduct all of the tests of PG species reported above in one single set of experiments. Likewise, it was impossible to treat all keratinocytes at exactly the same degree of confluence across the multiple experiments required, as the primary cultures were prepared from neonatal mice of slightly different ages resulting in somewhat different plating efficiencies and thus confluence at treatment. Because epidermal keratinocytes are contact inhibited, these differences in confluence likely resulted in the observed disparate proliferation under control conditions. Consequently, to allow comparison among all the experiments, egg PG was tested as a comparator in each experiment. Note that a total of 38 separate experiments from 38 different keratinocyte preparations/primary cultures were performed, each with its own egg PG control to allow comparison among experiments with different degrees of confluence. Based on the response to egg PG, all of the results could be grouped into four clusters as shown in Figure 4: (1) cluster 1 represented the experiments in which the response to egg PG was stimulatory, and in which the cells were slowly dividing, as indicated by the lower value at the zero concentration on the x-axis compared with those of clusters 2, 3, and 4; (2) cluster 2 represented the experiments in which the response to egg PG was monophasically inhibitory; (3) cluster 3 represented the experiments in which the response to egg PG was relatively flat; and (4) cluster 4 represented the experiments in which the response to egg PG was biphasically inhibitory (Figure 4). We found that in cluster 1, the stimulatory effect of DOPG was significantly more potent than that of egg PG while DPPG was significantly less potent. In cluster 2, POPG, DSPG, and DOPG were significantly less inhibitory than egg PG, whereas DLPG, PAPG, and soy PG were significantly more potent than egg PG at inhibiting proliferation. In cluster 3, in which egg PG had little or no effect on keratinocyte proliferation, soy PG still exhibited a significant inhibitory effect, suggesting greater potency of soy PG. In cluster 4, PAPG’s inhibition was significantly more potent than that of egg PG, whereas DPPG, POPG, and DHPG were significantly less inhibitory (or slightly stimulatory) relative to egg PG. These results imply that the acyl groups of PG species play a key role in the effects of this phospholipid signal on keratinocyte proliferation. Because the fatty acid tails can be released from the phospholipids potentially allowing them to exert independent effects on cells, it was important to test whether the glycerol head group, and thus the intact phospholipid, mediates the ability of PG species to modulate keratinocyte proliferation.

### Comparison of the effects of DLPG and DLPP on keratinocyte proliferation

In a previous study, we used liposomes composed of dioleoyl- or dipalmitoyl-phosphatidylpropanol (DOPP or DPPP) as a phospholipid control for PG [8]. Note that phosphatidylpropanol has essentially the same structure as PG, lacking only the two hydroxyl groups at the C2 and C3 positions of the head group. This makes it a good control for determining effects mediated by the glycerol head group. Comparison of the previous study with the present study indicates that the glycerol head group plays a role in the stimulatory effect of PG species containing saturated or monounsaturated fatty acids. Specifically, whereas DPPG tended to increase keratinocyte proliferation and DOPG significantly increased keratinocyte proliferation in the present study (Figure 1), neither DPPP nor DOPP had a significant effect on keratinocyte proliferation in the previous study [8]. To investigate whether the glycerol head group plays a role in the inhibitory effect of PG species containing polyunsaturated fatty acids, we compared the effects of DLPG and DLPP on keratinocyte proliferation. DLPG was selected because it was one of the most effective inhibitory PG species in the present study (Figure 2).

**Figure 5:**
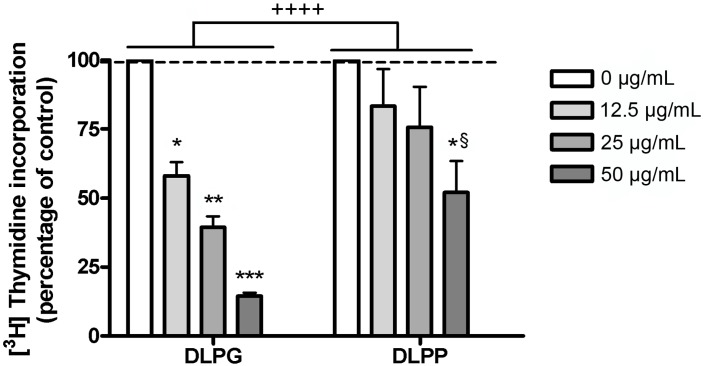
Comparison of the Effects of DLPG and DLPP on Keratinocyte Proliferation. Near-confluent to confluent keratinocytes were treated for 24 hrs with the indicated concentrations of liposomes composed of DLPG or DLPP, prepared via bath sonication of the phopholipid in SFKM. [^3^H]Thymidine incorporation into DNA was then determined. Values represent the means ± SEM of more than 3 separate experiments performed in duplicate; **p<0.01, ***p<0.001 versus the control value (0 µg/mL); §p<0.05 versus the corresponding concentration of DLPG; ++++ p<0.0001 as indicated. [^3^H]Thymidine incorporation into DNA in the control was 50,400±7,500 cpm/well, and 53,300±6,400 cpm/well for DLPG and DLPP, respectively.

As shown in Figure 5, both DLPG and DLPP, with the same polyunsaturated fatty acid tails, significantly inhibited keratinocyte proliferation compared with control. However, DLPG was more potent, exerting a significantly greater inhibitory effect than DLPP. In particular, DLPP induced a significant inhibition only at 50 µg/mL, and this dose was significantly less inhibitory than that of the corresponding dose of DLPG, whereas DLPG induced significant inhibition at one quarter the dose (12.5 µg/mL). These results suggest that the polar head group of PG does indeed play a role in the effect of PG species on keratinocyte proliferation, whether stimulatory or inhibitory, and argue against the idea that the effect of PG is mediated to any great extent through fatty acids released from the phospholipid.

## Discussion

PG is garnering attention as a physiologically active phospholipid with potential involvement in cell signaling. Older studies from the Elias laboratory suggested the existence of a PG-activated protein kinase (PK-P) in human leukemia cells and human spleen [15], [16]. A subsequent study indicated that this protein kinase was in fact, protein kinase C (PKC)-θ, which mediates the phosphorylation of the actin-binding sequence of moesin [17]. Similarly, Fields and colleagues published several reports [18]–[20] detailing the discovery of the role of nuclear PG in activating PKCßII to induce the phosphorylation of lamin B and the subsequent dissolution of the nuclear membrane during cell division in leukemia cell lines. More recent studies have suggested a role for PG in stabilizing membrane proteins [21], activating the ferlin family of proteins that mediate membrane trafficking [22], enhancing protein-protein interaction [23], [24] and protecting cells from the harmful effects of mitochondrial cardiolipin deficiency [25].

Our previous study provided evidence for the existence in primary mouse keratinocytes of a novel lipid signaling pathway, for which PG is a key effector in regulating keratinocyte proliferation and differentiation (reviewed in [26]). In particular, we showed that egg PG inhibits keratinocyte proliferation in rapidly dividing keratinocytes and stimulates keratinocyte proliferation in slowly dividing keratinocytes [8]. Since there are many species of PG, with different fatty acid compositions, present in egg PG and in the human body, in this report we sought to identify the most effective PG species able to normalize keratinocyte function, with the idea that this phospholipid could be used to treat skin diseases characterized by excessive or insufficient proliferation.

Unexpectedly, we found that PG with different compositions, that is, monounsaturated versus polyunsaturated fatty acid-containing species, had different effects on keratinocyte proliferation. In detail, PG species containing monounsaturated fatty acids, such as the oleic acid in POPG (16∶0/18∶1) and DOPG (18∶1/18∶1), stimulated mouse keratinocyte proliferation, while PG species containing polyunsaturated fatty acids, such as the arachidonic and linoleic acids in PAPG (16∶0/20∶4), PLPG (16∶0/18∶2) and DLPG (18∶2/18∶2), inhibited mouse keratinocyte proliferation. It is perhaps not surprising that PG species with different acyl groups have different signaling functions since in the lung saturated PG cannot block the anti-inflammatory effects of surfactant protein A on lipopolysaccharide (LPS)-treated macrophages while unsaturated PG can [27]. Nevertheless, to our knowledge, ours represents the first report of opposite effects of two species within the same phospholipid class on a particular cellular response. Our previous studies suggested that PG liposomes might be an ideal treatment to normalize skin function under both pathological and physiological conditions [8]. The discovery reported here suggests that specific PG species might be used under different conditions.

The efficacy of linoleic acid-containing PGs is intriguing considering the fact that this fatty acid is the predominant species in the epidermis. Thus, Marcello and colleagues found that linoleic acid represents over 20% of the fatty acid species in the epidermis [28]. Interestingly, linoleic acid percentage was higher in the suprabasal layers of the epidermis (27.4%) in comparison with the basal layer (20.7%). In addition, polyunsaturated fatty acids compose 37% of the fatty acids of suprabasal epidermis [28]. This result suggests the possibility that PGs containing polyunsaturated fatty acids are physiologically relevant in terms of regulating keratinocyte proliferation. Furthermore, it would suggest that PG formed from the action of PLD2 on phosphatidylcholine in the presence of glycerol (provided by AQP3) may contain a high proportion of polyunsaturated fatty acids.

For each PG species we tested, we also tested at the same time within the same cell preparation the effects of egg PG as a comparator. By performing an egg PG dose response in all experiments, a later assessment among experiments could be accomplished. As demonstrated previously [8], we observed both inhibitory and stimulatory effects of this phospholipid on keratinocyte proliferation (Figure 4). Egg PG exhibited an inhibitory effect on proliferation in rapidly proliferating cells (clusters 2–4 of Figure 4), while in slowly proliferating keratinocytes, egg PG showed stimulatory effects on cell proliferation (cluster 1 of Figure 4). This result suggested that under different physiologic or pathologic conditions, specific PG species can exert opposite effects to “normalize” keratinocyte proliferation, although the current report suggests that this normalization reflects, at least in part the presence in egg PG of more than one PG species with different signaling functions.

Although synthetic polyunsaturated fatty acid-containing PGs, and in particular DLPG, seemed most effective at inhibiting keratinocyte proliferation *in vitro*, the expense of these PGs could potentally preclude their use as a treatment for psoriasis. Therefore, we also investigated the ability of soy PG, a mixture of PG species containing a high proportion of polyunsaturated fatty acids, to inhibit keratinocyte proliferation. This lipid also has the advantage of being a natural product, and our results *in vitro* indicated its efficacy (Figure 3).

Previously, we had also shown that neither DOPP nor DPPP altered keratinocyte proliferation [8]; however, the corresponding PG species either tended to stimulate keratinocyte proliferation (DPPG) or significantly stimulated keratinocyte proliferation (DOPG). Likewise, although both DLPG and DLPP (18∶2/18∶2) inhibited keratinocyte proliferation, the effect of DLPG was significantly greater than that of DLPP. Together these results indicate the importance of the head group in determining the effects of PG and argue against the idea that the fatty acids are being released from the PG phospholipid to induce the disparate effects observed.

Several questions remain. For example, what downstream targets of the specific PG species exert the inhibitory or stimulatory effects on keratinocyte proliferation? We speculate that PG exerts different effects through different effector pathways. One potential mechanism stems from the observation by Murray and Fields [18] that in human leukemia cells PG binds to and stimulates protein kinase CßII (PKCßII), an important protein kinase mediating proliferation in these cells. Furthermore, these authors demonstrated that specific PG species exhibit different activities but that other phospholipids (phosphatidylserine and phosphatidylcholine) do not mimic the effect of PG, suggesting that the ability of PG to activate PKCßII resides in the head group [18]. A role for the fatty acid side chain constituents is also provided by the finding that DOPG was significantly more potent at stimulating PKCßII activity than the other PG species tested [18]. Likewise, DOPG produced a potent stimulatory effect on DNA synthesis, suggesting the possibility that PKCßII might underlie the growth effects of PG [18]. On the other hand, many PKC isoforms trigger differentiation rather than proliferation in keratinocytes [29], [30]. Moreover, Murray and Fields [18] did not determine the efficacy of PG species containing polyunsaturated fatty acids (the only species tested were DOPG, DPPG, POPG, and DSPG). Therefore, it is possible that PKCßII stimulated by polyunsaturated fatty acid-containing PG may instead be prodifferentiative in keratinocytes. Indeed, in other experiments we have determined that overexpression of PKCßII appears to sensitize keratinocytes to the differentiating effects of PG as well as of an elevated extracellular calcium concentration (manuscript in preparation), which stimulates the production of PG [5].

PG might also function by an ability to modify pattern recognition receptor signaling, such as occurs upon activation of toll-like receptors (TLR). Pattern recognition receptors can be activated not only in reponse to pathogen-associated molecular patterns but also damage-associated molecular patterns arising from cell injury (reviewed in [31], [32]). For example, PG, produced by alveolar cells as a significant component of pulmonary surfactant, inhibits TLR signaling in macrophages exposed to LPS *in vitro*, acting at multiple sites to disrupt TLR4 signaling [33], as well as TLR2 pathway activation in response to bacterial and mycoplasma byproducts *in vitro* [34]. PG also protects the lungs from inflammation initiated by LPS exposure *in vivo* [33]. In addition, PG inhibits infection of airway epithelial cells by respiratory syncytial virus and influenza A *in vitro* and protects the lungs from the deleterious effects of these viruses *in vivo* [35], [36]. Finally, in eukaryotes PG is a precursor of cardiolipin [37], a key mitochondrial lipid that plays a role in mitochondrial function and energy production as well as apoptosis (reviewed in [38]). Indeed, PG can partially substitute for cardiolipin in several cellular functions [25]. Moreover, PG can inhibit cell death in response to apoptosis-inducing agents and/or cardiolipin deficiency [25], [39], suggesting another possible mechanism by which this lipid signal might regulate keratinocyte growth and differentiation.

In summary, we showed that PG species containing polyunsaturated fatty acids were effective at inhibiting rapidly proliferating keratinocytes, whereas PG species with monounsaturated and saturated fatty acids were effective at promoting proliferation in slowly dividing keratinocytes. Our results support the idea that these effects require both the glycerol headgroup and the fatty acid tails because (1) different fatty acids had disparate actions and (2) phospholipids similar to PG but lacking the glycerol headgroup (phosphatidypropanol) were less efficacious than the corresponding PG in promoting proliferation or differentiation. To our knowledge these findings also represent the first (and so far only) demonstration of the ability of different species within a given phospholipid class to induce opposite effects in intact cells. Future investigation is ongoing to determine the downstream targets of the AQP3-PLD2-PG signaling pathway and the mechanism by which the different PGs exert these disparate effects. Since abnormal keratinocyte proliferation and differentiation characterize a wide range of skin diseases, the findings of this study may open new avenues for treatment of these diseases. Furthermore, our results suggest that different PG species may be useful for treating various skin diseases characterized by excessive or insufficient proliferation.
